# Defect-induced B_4_C electrodes for high energy density supercapacitor devices

**DOI:** 10.1038/s41598-021-90878-0

**Published:** 2021-06-02

**Authors:** Özge Balcı, Merve Buldu, Ameen Uddin Ammar, Kamil Kiraz, Mehmet Somer, Emre Erdem

**Affiliations:** 1 Koç University Boron and Advanced Materials Application and Research Center, Rumelifeneri Yolu, 34450 Sarıyer, İstanbul, Turkey; 2 Department of Chemistry, Koç University, 34450 Sarıyer, İstanbul, Turkey; 3 Faculty of Engineering and Natural Sciences, Sabanci University, 34956 Tuzla, İstanbul, Turkey; 4 Pavezyum Chemicals Inc., Tuzla, İstanbul, Turkey; 5 Integrated Manufacturing Technologies Research and Application Center and Composite Technologies Center of Excellence, Sabanci University, Teknopark Istanbul, 34906 Pendik, İstanbul, Turkey

**Keywords:** Materials for devices, Physical chemistry, Energy

## Abstract

Boron carbide powders were synthesized by mechanically activated annealing process using anhydrous boron oxide (B_2_O_3_) and varying carbon (C) sources such as graphite and activated carbon: The precursors were mechanically activated for different times in a high energy ball mill and reacted in an induction furnace. According to the Raman analyses of the carbon sources, the I(D)/I(G) ratio increased from ~ 0.25 to ~ 0.99, as the carbon material changed from graphite to active carbon, indicating the highly defected and disordered structure of active carbon. Complementary advanced EPR analysis of defect centers in B_4_C revealed that the intrinsic defects play a major role in the electrochemical performance of the supercapacitor device once they have an electrode component made of bare B_4_C. Depending on the starting material and synthesis conditions the conductivity, energy, and power density, as well as capacity, can be controlled hence high-performance supercapacitor devices can be produced.

## Introduction

Boron carbide (B_4_C) is one of the hardest materials available to be used for commercial applications. The utilization of it in high-performance applications is due to its remarkable properties such as high hardness, high melting point, high elastic modulus, low density, and high neutron absorption cross-section^1^. B_4_C is a highly demanding refractory material used as controlling rods, shielding material, and neutron detector in nuclear reactors due to its excellent neutron absorbing ability^2,3^. As the major feature of B_4_C is its hardness, it found also applications in making body and vehicle armor^4^, cutting tools of different alloys^5^, and blasting nozzles specifically in the sintered form^6^. B_4_C has shown great potential to be used as a primary material in the electronic industry since it is a p-type semiconductor^7^. It is used in thermocouples, transistor devices, diodes and, thermoelectric material owing to its high Seebeck coefficient^8^. Thin films of B_4_C are used as a protective coating in electronic industries^9^.

The composition of the boron carbide, with a stoichiometric formula of B_4_C, can vary from carbon-rich (B_4.3_C) to boron-rich (B_~10.8_C) by partial substitution of B by C atoms. In the stable phase, the carbon concentration is generally from 8 to 20 at.%, however, a single crystal of boron carbide (B_11.4_C_3.6_ or B_~3.2_C) having 24 at.% C concentration is also reported^55^. Although the exact phase compositions, phase stabilities, and the formation mechanisms of defects are not fully understood yet, available experimental and computational data suggest a rhombohedral structure consists of 15 atoms per elementary cell with slightly distorted 12-atom icosahedra of B_12_, B_11_C, or B_10_C_2_ located at the corners which linked by a central three-atom linear chain of C–C–C, C–B–C or C–B–B, or chains with vacancies^10,11^. Among them, the hypothetical compound B_12_ (C–B–C), which is the idealized, energetically most favorable composition of boron carbide, is preferred for theoretical calculations^12^. On the other hand, stoichiometric B_4_C with lattice parameters of *a* = 5.16 Å and α = 65.7° is the preferred structure for commercial use. However, the literature also reports the hexagonal lattice representation of B_4_C with parameters of *a* = 5.60 Å, *c* = 12.07 Å, and an axial ratio of *c/a* = 2.155^5^.

A perfect structure of boron carbide as depicted above is very difficult to achieve as the synthesis of B_4_C occurs at very high temperatures along with the solute’s increased chemical activities^13^. So, the reason for the formation of potential defect sites in boron carbide are (1) Impurities in initial boron carbide components (b) localized states produced in the sample preparation process, (c) the heterogeneity of component distribution over the bulk of the sample, (d) granular occurrence formed because of the hot-pressing of crystallites, (e) development of native defects in boron carbide lattice^14^.

For designing efficient devices based on B_4_C, an atomistic-level understanding of nature and formation mechanism of possible planar and point defects in B_4_C is necessary as defects are one of the most important factors affecting the mechanical, thermal, optical, and electrical properties of the materials. Different types of defects in boron carbide can be introduced during the manufacturing process^15,16^, may form during the heat treatment of the samples^17,18^, can be generated under specific operating conditions where the samples subjected to high pressure and shear deformation^19–22^ or upon irradiation with neutrons or different wavelengths of light^23–27^.

Heian et al*.* synthesized a highly defective nanostructured B_4_C through ball-milling of stoichiometric mixtures of amorphous boron and carbon and pressed under an argon atmosphere at high temperatures^16^. Contrasting results on the size of synthesized crystallites obtained with TEM and XRD analyses were attributed to the presence of possible high density of twins present in the B_4_C structures^16^. Ektarawong et al*.* carried out first-principles calculations to investigate the effect of high pressure on the structural stability of B_4_C^28^. The study showed that the orientational ordering transition temperature of B_4_C increases with pressure, and the electronic properties of B_4_C, i.e., bandgap, is affected by the configurational disorder. Their findings also suggest that the increase in bandgap with increased pressure may be due to the decreasing concentration of high-energy defects^28^.

Detailed microscopic and spectroscopic analyses along with ab-initio calculations have been utilized to characterize the possible defects in boron carbides^28–32^. Even though theoretical predictions assume a perfect structure with metallic character, experimental results proved that B_4_C is a p-type semiconductor with a defect concentration of 1–10% or more^33^. The main reason for this discrepancy between experimental results and theoretical calculations is the high concentration of intrinsic point defects in the structure^34^. Due to the unique rhombohedral structure of B_4_C, there are many interstitial sites available in the structure that can accommodate valences and interstitial atoms, which are very common point defects in B_4_C^11^. The three-atom chains are suggested as preferred locations for vacancy accumulation which initiates the formation of C–C bond under high pressure and stress, resulting in shear deformation along the plane due to the weakened bonding between the C atoms forming the chain^19,35^.

Transition metal carbide based materials has been recently showed good performance in electrochemical energy storage mechanisms in particular in the field of batteries and capacitors^36^. Molybdenum, titanium and iron based carbides Mo_2_C, TiC, and Fe_3_C, respectively has been recently used as electrode because of their outstanding electrical conductivities. In specific, Gogotsi et al*.* developed enormous amount of supercapacitors by the aid of MXenes materials which uses mostly Ti_3_C_2_ based materials systems^37^. Our joint experiences on the development of metal oxide based battery-like pseudo supercapacitors and metal boride/carbide based advanced materials motivated us to search the capacitive behavior of B4C compound^38^. Similar achievements can be obtained for B_4_C as well once their local electronic structures and defects are controlled. Such studies are very less in literature so that it is highly worthy to test the non-metallic carbide B_4_C as supercapacitor electrode. For instance, SiC nanowires has been used as electrode for the production of micro-supercapacitors and a very high specific capacitance values has been obtained up to 240 μF/cm^2^^39^.

In the light of limited literature data that are so far published, in this work the defect structures will be mainly investigated via EPR spectroscopy. The concentration of defect centers will be accurately determined with the help of an analytical spin counting procedure. Finally, the most defective sample will be tested in terms of its electrochemical performance and finally, the features and prospects of B_4_C materials in energy storage systems will be discussed.

## Experimental

### Synthesis

Boron carbide powders were synthesized by mechanically activated annealing method by using anhydrous boron oxide (B_2_O_3_) and varying carbon (C) sources such as graphite and activated carbon. In the mechanically activated annealing (M2A) process, high energy milling provides the mechanical activation of the powder particles and subsequent annealing enables the activated particles to react at lower temperatures than thermodynamically required^40,41^. In this study, a mechanical activation assisted carbothermal reduction process was used to obtain boron carbide powders which are based on the main principles of M2A routes. Stoichiometric amounts of B_2_O_3_ (ETI Mine, 98% purity, 250 µm average particle size) and C powders were weighed according to the theoretical formation reaction given in Eq. (1) in order to constitute powder batches of 6 g.

1 $$2{\text{B}}_{2} {\text{O}}_{{3{\text{(s)}}}} + 7{\text{C}}_{{\text{(s)}}} \to {\text{B}}_{4} {\text{C}}_{{\text{(s)}}} + 6{\text{CO}}_{{\text{(g)}}}$$

**Figure 1:**
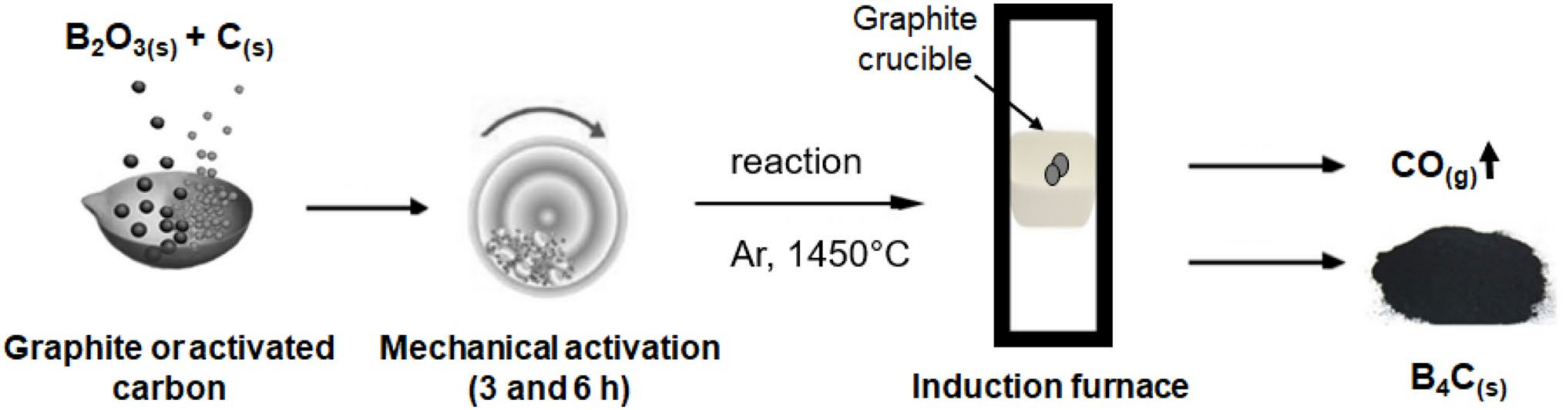
Schematic representation of the B_4_C synthesis experiment.

Powder mixtures prepared with graphite (Merck, < 50 μm particle size) or activated carbon (Merck, < 30 μm particle size) are mechanically activated in a high energy ball mill (Retsch) operated at 350 rpm for different times of 3 and 6 h. Ball-to-powder weight ratio (BPR) of 4:1 was used during all milling experiments. Sample handling was carried out in a glove box (MBraun) under a purified Ar atmosphere to prevent surface oxidation and contamination of powders from atmospheric conditions. Mechanically activated powders were pelletized in a cold hydraulic press under a uniaxial pressure of 300 MPa. The pellets were annealed in an induction furnace (MTI) under flowing Ar gas atmosphere at 1450 °C for 6 h. The annealing process was performed in a graphite crucible placed in a silica tube. The actual temperature inside the reaction region was measured by a pyrometer in the direct vicinity of the sample center. After the reaction, the pellets are grounded in an agate mortar to obtain the final powders. The schematic representation of the synthesis experiment is given in Fig. 1.

Table 1 summarizes the sample names and their synthesis conditions. The reacted products, which were prepared by using graphite or activated carbon source, were consisted of pure B_4_C phase containing 13 and 4% remaining carbon, respectively. Thus, the usage of activated carbon as a carbon source enabled to reduce the remained carbon phase in the resulting powders. Furthermore, the average particle size of the synthesized B_4_C powders was approximately 10 µm.

**Table 1:** B_4_C powders synthesized using anhydrous boron oxide (B_2_O_3_) and varying carbon (C) sources.

Sample name	Synthesis conditions		
	Carbon source (C)	Milling time (h)	Reaction
S1@G3	Graphite	3	Induction furnace (at 1450 °C for 6 h)
S2@G6	Graphite	6	
S3@A3	Active carbon	3	
S4@A6	Active carbon	6	

### Methods

The phase analysis of the powders was performed by an X-ray diffractometer (Bruker D8 Advance, XRD) using CuK_α_ radiation. Microstructural characterizations were performed by using a Zeiss Ultra Plus Field Emission Scanning Electron Microscope (FE-SEM) coupled with an energy dispersive X-Ray spectrometer (EDS). Secondary electron detector was used to obtain the images (10 kV of acceleration voltage) by setting the working distance as about 10 mm. Chemical analyses were done by using an organic element analyzer (Thermo Scientific Flash 2000) to calculate the total and remained carbon quantitatively. Differential thermal analysis/thermogravimetric (DTA/TG, STA449F3, Netzsch) apparatuses were used to carry out the thermal analyses, which were performed using an alumina crucible up to the temperature of 1200 °C with a 10 K/min heating rate under Ar atmosphere.

**Figure 2:**
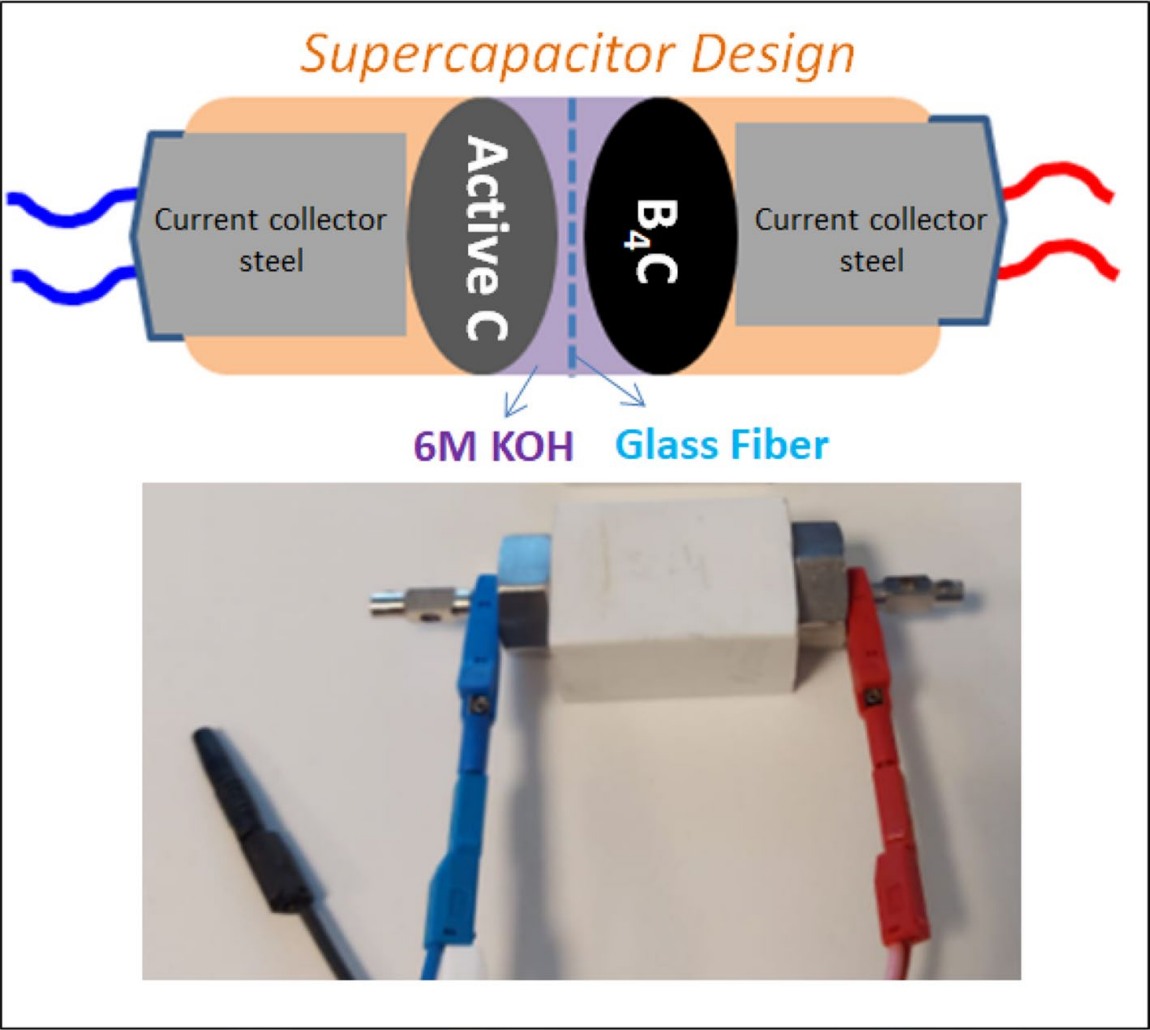
Design of the supercapacitor device and its real photograph while it is testing via potentiostat.

For the structure/surface analysis of the graphite and active carbon powders, the Raman spectroscopy (Renishaw Invia) technique was utilized. An excitation laser source of 532 nm was used during the analysis at room temperature. The laser power was kept at 0.1 mW to avoid local heating and phase transformation through oxidation. EPR measurements were performed with the spectrometer of Bruker EMX Nano with an integrated referencing for g-factor calculation and also integrated spin counting units. The microwave frequency of the cavity was 9.41 GHz (X-band) and all spectra were measured at room temperature with 2 G modulation amplitude, 1 mW microwave power, and 10 scans each scan has sweep time and time constant of 120 s and 81.92 ms, respectively. Samples were inserted into spin-free 25 cm long quartz tubes (Qsil, Germany). The electrochemical performance tests were performed on BioLogic VMP 300 electrochemical workstation having electrochemical impedance spectroscopy (EIS) channel unit. All electrical tests were made at room temperature using 6 M KOH as the electrolyte. Whatman glass microfibers were used as the separator. Electrochemical properties were examined using potentiostatic electrochemical impedance spectroscopy (PEIS). PEIS results were accomplished by applying a sinusoidal signal of 10 mV from 10 mHz to 1 MHz frequency range. Galvanostatic cycling with Potential Limitation technique (GCPL) was recorded at a scan rate of (10 mV/s) within voltage window − 1 to + 1 V at a specific current of 0.1 A/g. Further (given in Supp Material) various specific current has been applied at 0.1 A/g, 0.2 A/g, 0.3 A/g, 0.5 A/g, 2.5 A/g and finally back to 0.15 A/g to test the stability. The supercapacitor device design has been done as follows: Electrode1: One of the B_4_C samples (S1@G3, S1@G6, S3@A3, S4@A6), electrode2: active C, electrolyte: 1 M KOH, and separator: glass fiber. Traditional two point probe method has been applied to test the devices. Here there is no binder or any additive has been used to prepare the electrodes. Powder electrodes have been inserted together with electrolyte and separator into the mounting devices and two electrodes become a pellet form inside the mounting device separated by glass fiber. To illustrate the elements each element was shown separately in Fig. 2 for helping the eyes. Note that in reality, the electrodes are touching the surface of the separator.

## Results and discussions

**Figure 3:**
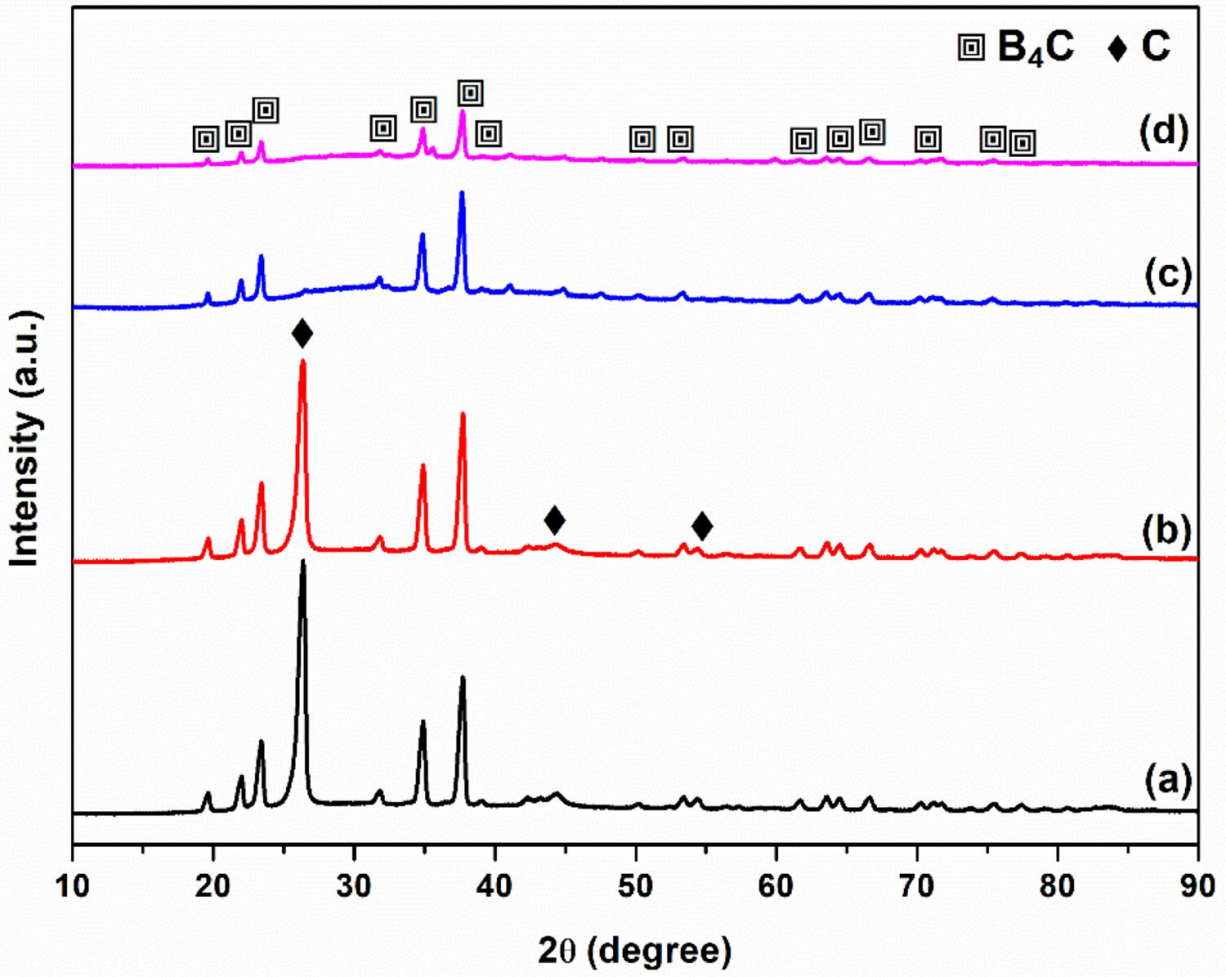
XRD patterns of the synthesized B_4_C powders: (**a**) S1@G3, (**b**) S2@G6, (**c**) S3@A3, and (**d**) S4@A6.

For microstructural understanding of the present samples XRD and FE-SEM equipped with EDS unit has been used. Figure 3 shows the XRD patterns of the synthesized B_4_C powders. For comparison purpose, the XRD patterns of the precursors after mechanical activation are given in Figure S1 (In Supporting Information File). As we compare the XRD analyses in Figure S1 and 2, it is clear that mechanically activated powders containing anhydrous boron oxide (B_2_O_3_) and varying carbon (C) phases were successfully reacted at 1450 °C and resulted in crystalline B_4_C phase (Boron carbide, ICDD Card number: 01-086-1118, crystal system: rhombodehral, *a* = *b* = 5.6274 Å, *c* = 12.1472 Å) in the all powders. Mechanical milling of B_2_O_3_ and active C (both for 3 and 6 h) resulted in complete amorphization of C phase, as no crystalline peak of C was detected in the mechanically activated powders in Figure S1(c) and (d). Furthermore, there is an observable decrease in the intensities of B_2_O_3_ peaks from 3 to 6 h of milling times (Figure S1). Previous studies done on the milling for short times (≤ 1–2 h) showed that at least 3 h of milling is required for a sufficient amorphization, which later provided a complete conversion of B_2_O_3_ to B_4_C (Fig. 3c, d). On the other hand, crystalline C phase (Graphite, ICDD Card number: 01-075-1621, crystal system: hexagonal, *a* = *b* = 2.4700 Å, *c* = 6.7900 Å) was detected in the XRD patterns of the powders synthesized using graphite (Fig. 3a, b), indicating the unreacted C in the resulting powders. The high intensity of C phase is most likely due to the further crystallization of unreacted graphite during long period of annealing (6 h). Chemical analyses showed that the reacted products, which were prepared by using graphite or activated carbon source, were consisted of pure B_4_C phase containing 13 and 4% remaining carbon, respectively. Thus, the usage of activated carbon as a carbon source enabled to reduce the remained carbon phase in the resulting powders.

SEM images of the synthesized B_4_C powders are given in Figures S2 and S3 (In Supporting Information File). Figures S2a and S2b present the different morphologies of crystalline B_4_C and C phases (analyzed by EDS point analyses) in the microstructure of the powders synthesized by using the graphite as carbon source. On the other hand, the powders prepared by active carbon did not reveal any carbon structure (Figures S2c and S2d). The SEM images at higher magnifications clearly show the microstructural difference between the powders synthesized by using graphite or active carbon (Figure S3). These observations are also consistent with the chemical and phase analyses of the samples indicating high and low amount of remaining carbon in the S1@G3/S2@G6 and S3@A3/S4@A6 samples, respectively. Furthermore, the average particle size of the synthesized B_4_C powders was approximately 10 µm. In summary, the S1@G3 and S2@G6 samples are the powders containing B_4_C phase with an amount of 13 wt.% of C phase, whereas the S3@A3 and S4@A6 samples are ones containing pure B_4_C phase with a slight amount of C (not detected in XRD analyses).

To show the thermal stability with respect to different milling times, thermogravimetric analyses (TGA) were performed on the powders containing low amount of free C, which were synthesized by using active carbon. Figure S4 shows the TGA of the synthesized B_4_C powders, which were performed up to 1200 °C under Ar atmosphere. Powders synthesized using 3 h of milling time showed a mass decrease at about 950 °C, indicating the evaporation of remained boron oxide (9% of decrease in total mass). On the other hand, powders synthesized using 6 h of milling time did not show any mass loss, indicating a very high thermal stability. Thus, increasing the milling time from 3 to 6 h provided to enhance the thermal stability from 950 to 1200 °C.

EPR spectroscopic technique is very well suited for understanding the role of native point defects since it provides a direct way to observe various paramagnetic defect states. Thus, it complements other experimental methods that are giving information on the electronic structure such as Raman spectroscopy. Combined studies of Raman and EPR spectroscopy always give extensive information on the defect structures in particular for SiCN^42^, MgB_2_^43,44^, ZnO^45^, and C-dots^46,47^. On the other hand, in B_4_C sample systems, due to lack of analytical characterization such as data from advanced EPR techniques, which preclude the unambiguous determination of defect states, the role of native defects on B_4_C properties remains unclear and is still a matter of debate. This is mainly due to the fact that at present most of the researchers who investigate B_4_C properties are trying to improve phase stability while overlooking the effect of defect structures at a microscopic level. For instance, one of the carbon inclusions in polycrystalline B_4_C studies by EPR unambiguously showed that point defects played a significant role in achieving a much higher surface-to-volume ratio by the inclusion of free C at the surface layer of the material^48^. In another study, the dependence on temperature and thermal treatment of the B_4_C powders were studied in detail by EPR spectroscopy by just probing the intrinsic defect structures. It has been demonstrated that native defects of B_4_C and conduction electrons are responsible for EPR absorption^13^.

**Figure 4:**
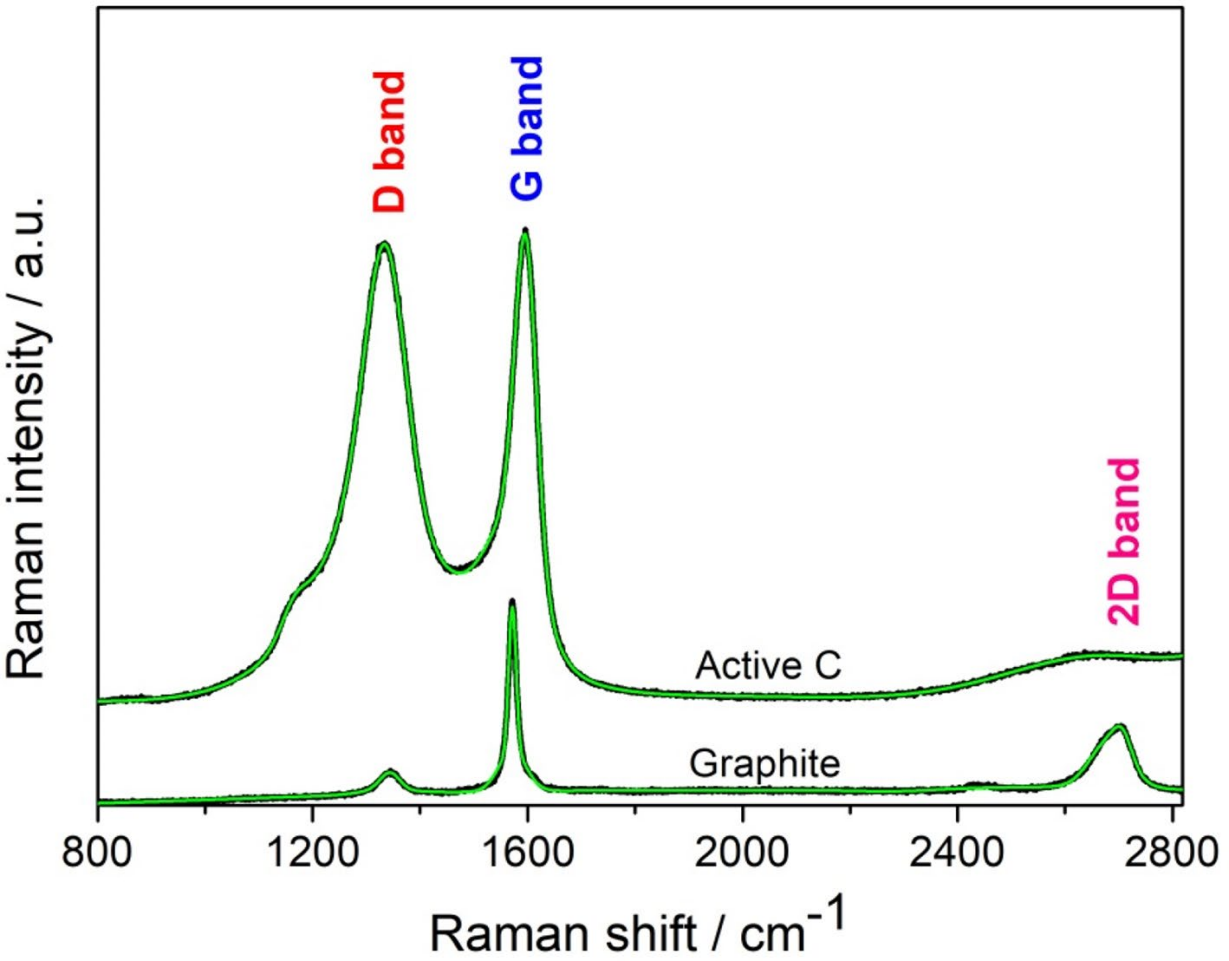
Raman spectra of graphite and active C materials with their Gaussian fits (green) in order to determine the band ratio.

As a result, in the enlightenment of previous studies, we employed here both Raman and EPR spectroscopy and deduced the valuable information as follows: Fig. 4 presents the Raman spectra of the graphite and active C raw materials. The first peak is the D-band (pseudo-Voigt) around 1340 cm^−1^, a defect-induced Raman mode of graphite that is not observed in perfect graphitic structures^49^. The G-band (pseudo-Voigt) around 1580 cm^−1^ belongs to defect-free graphite^50^. There are three peaks in the Raman spectra of graphite presented in the wavenumber range of 100 to 3000 cm^−1^, which correspond to D- (~ 1344 cm^−1^), G- (~ 1571 cm^−1^), and 2D- (sometimes called G*'*) (~ 2700 cm^−1^) bands, respectively. The G-band appeared at a slightly higher wavenumber (~ 1592 cm^−1^) in the spectra of active carbon than in graphite. This shift may result from the crystallite size differences between different carbon domains.

The intensities of these bands are related to the amount of graphitization degree, where the intensity of D-band is proportional to the amount of disordered *sp*^3^ carbon, and the intensity of the G-band is proportional to the amount of ordered graphitic (*sp*^2^) carbon contained in the sample. The 2D-band is also related to the amount of disordered *sp*^3^ carbon, but its intensity usually affects the property of the used laser. The 2D-band around 2700 cm^−1^ was not observed in the spectra of active carbon (Fig. 4). Since this is related to the performance or intensity of the used laser, 2D-band peak is not used for analyzing the hybridization behavior or conductivity of the samples. Therefore, we compared here the intensities of the two characteristic main peaks of D- and G-bands, to determine the graphitization degree of two different carbon precursors.

The D-band, which arises from the defects and disorders in the carbon lattice, significantly increased in the Raman spectra of active carbon^51^. Based on the origins of the D- and G-bands, the intensity ratio of the D- to G-band (I(D)/I(G) ratio) can be used to estimate the defect density of carbon materials^51^. According to the Raman spectra in Fig. 4, the I(D)/I(G) ratio increased from ~ 0.25 to ~ 0.99, as the carbon material changed from graphite to active carbon. The high I(D)/I(G) ratio in the spectra of active carbon proves its highly defected and disordered structure. On the other hand, a low I(D)/I(G) ratio in the spectra of graphite would mean the graphitization is high, which might be lead to a better electrical conductivity^47^.

**Figure 5:**
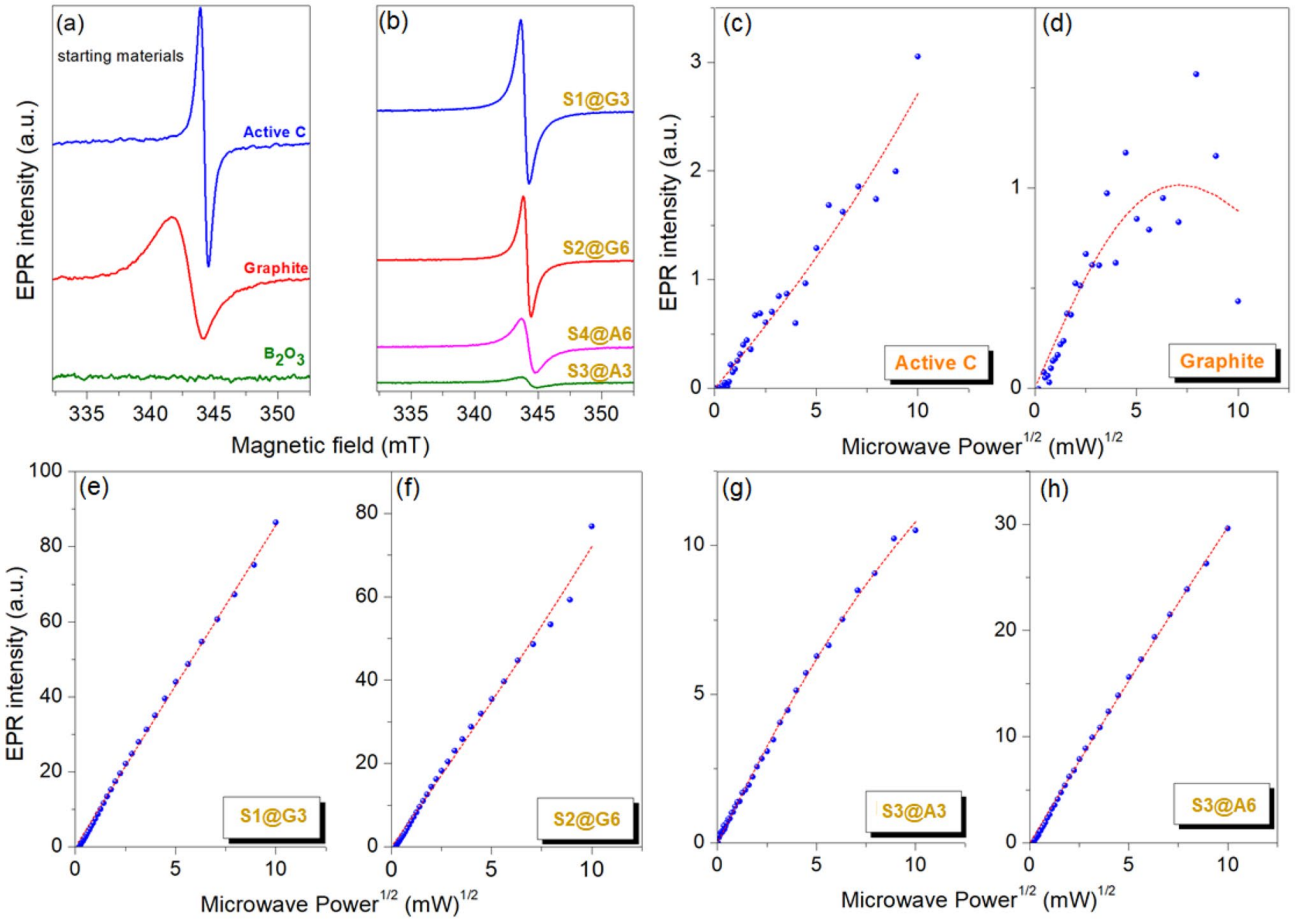
(**a**, **b**) X-band continuous wave EPR results and, (**c**–**h**) experimental and fitted EPR power saturation curves for starting materials and B_4_C samples.

EPR spectroscopy is one of the superior magnetic resonance techniques which give valuable information on the local electronic configuration in the crystal lattice. In particular, EPR is extremely sensitive to paramagnetic metal ions such as Fe^3+^, Mn^2+^, and Cr^3+^ or defect centers of diamagnetic materials where the electrons are trapped and become ionized hence paramagnetically active. In this context, materials like semiconductor ZnO^52^, superconductive MgB_2_^43^ and, high-dielectric Ta_2_O_5_^53^ show EPR active defect centers although they are diamagnetic. The formation energy of defect centers mainly determines the concentration of the defects that mainly exist in the material. The main defect structure discussion in B_4_C material is whether the EPR signal arises from the free-carbon or localized-carbon defects. The discussion in literature mainly focuses on these two main defect centers based on the interexchange of carbon and boron atoms and the possible existence of B_12_, B_11_C, and B_10_C_2_ icosahedra, as well as the permissibility of the C–B–B, C–B–C, and C–C–C chains. In the present work, high-grade B_2_O_3_ was used together with either Active C or graphite for obtaining B_4_C. The defect structures will be explained by monitoring the defects in starting materials, basically the carbonaceous ones. Finally, their defect contribution will be analyzed in B_4_C. In Fig. 5a, X-Band (9.64 GHz) room temperature EPR spectra are given for Active C, graphite, B_2_O_3,_ and the final products of B_4_C were presented. As expected, diamagnetic B_2_O_3_ in EPR inactive therefore no significant EPR signal was detected. This also shows that the B_2_O_3_ starting material is defect-free and does not possess any impurities. In general, high ceramic materials such as PbTiO_3_^54^, BaTiO_3_^55^, or PbZrTiO_3_^56^ are EPR inactive because their crystal field is too high (in the order of GHz) compared to Zeeman energy. For instance, the crystal field energy of ZnO is in the order of MHz and it always gives EPR signal due to intrinsic defect centers such as oxygen or zinc vacancies/interstitials^57^. On the other hand, carbonaceous materials of Active C and graphite both revealed EPR signal with completely different features. The *g*-factor and the EPR linewidth of both carbonaceous materials were different indicating that the defect kinds and their environment are different. Also, the EPR intensity of both materials is different indicating different concentrations. In the present case, active C and graphite have two distinct difference (i) the isotropic *g*-factor of active C is 2.0031 and graphite is 2.0098 (refer Table 2) and, (ii) the linewidth is much higher for graphitic carbon. Such distinctive features in EPR signal of defects reflect their intrinsic characteristics into the produced end product, B_4_C. The interesting EPR features can be understood in Fig. 5b in accordance with Table 2. By the aid of sophisticated spin counting procedures it is possible to determine the defect concentration from EPR spectrum. The details of spin counting procedure are given in Supp. Mater. Shortly, the area of doubly integrated EPR spectrum is directly proportional to the concentration of paramagnetic species. Hence, the highest defect concentration here has the S1@G3 sample which is based on graphitic carbon. Compared to other graphitic samples S1@G6 which is a longer milled S1@G3 sample has almost factor 2 higher defects. One of the best ways to understand the electronic properties of trapped electrons at the defect sites via EPR is to monitor their EPR intensity change by increasing the microwave (MW) power gradually. The square root of MW power versus intensity profiles which are given here in Fig. 5c–h is the key results to see whether the defects are contributing to the electrical conductivity or not. Such an approach has been already applied very effectively to ZnO and other kinds of materials^44,45^. According to the results obtained via microwave power saturation, except for the graphite sample, all other materials revealed unsaturated line shape. Graphite in Fig. 5d has a strong deviation from linear dependency and at around 25 mW starts to saturate. On the other hand, graphite and active C contains different species of paramagnetic defects hence they have completely different saturation behavior. On the other hand, all B_4_C material revealed an unsaturated curve which also indicates the same kind of defect center which is paramagnetic. In short, the physical meaning of saturated and unsaturated curves in EPR is as follows^52,58^. Easy saturated systems reveal Gaussian type lineshape indicating inhomogeneous broadening. Such species mostly consist of localized electrons and contribute to conduction whereas non-saturated systems have EPR line shape of Lorentzian that are mostly localized and give strong deviation from the free-electron g-factor which is 2.0023. As it is seen in the present case graphite shows both easy saturation and it has the most deviated g-factor which is 2.0098 as given in Table 2. Thus the B_4_C samples synthesized based on graphite will give higher conductivity. Hence we concentrated more to the sample of S1@G3 which is made of graphite and having the highest defect concentration according to spin counting. Therefore we have tested its performance in a supercapacitor device and present its electrochemical performance.

**Table 2:** *g*-factors, integrated area of EPR signal, and the accurate defect concentration of carbon-based starting materials and B_4_C samples synthesized via various conditions.

	*g*-factor (isotropic)	Integrated area	Defect concentration (spins/g)
Active C	2.0031	1.23 × 10^2^	2.15 × 10^17^
Graphite	2.0098	2.82 × 10^2^	4.93 × 10^17^
S1@G3	2.0023	4.81 × 10^3^	8.41 × 10^18^
S2@G6	2.0031	2.52 × 10^3^	4.41 × 10^18^
S3@A3	2.0025	5.36 × 10^1^	9.38 × 10^16^
S4@A6	2.0027	4 × 10^3^	7 × 10^18^

**Figure 6:**
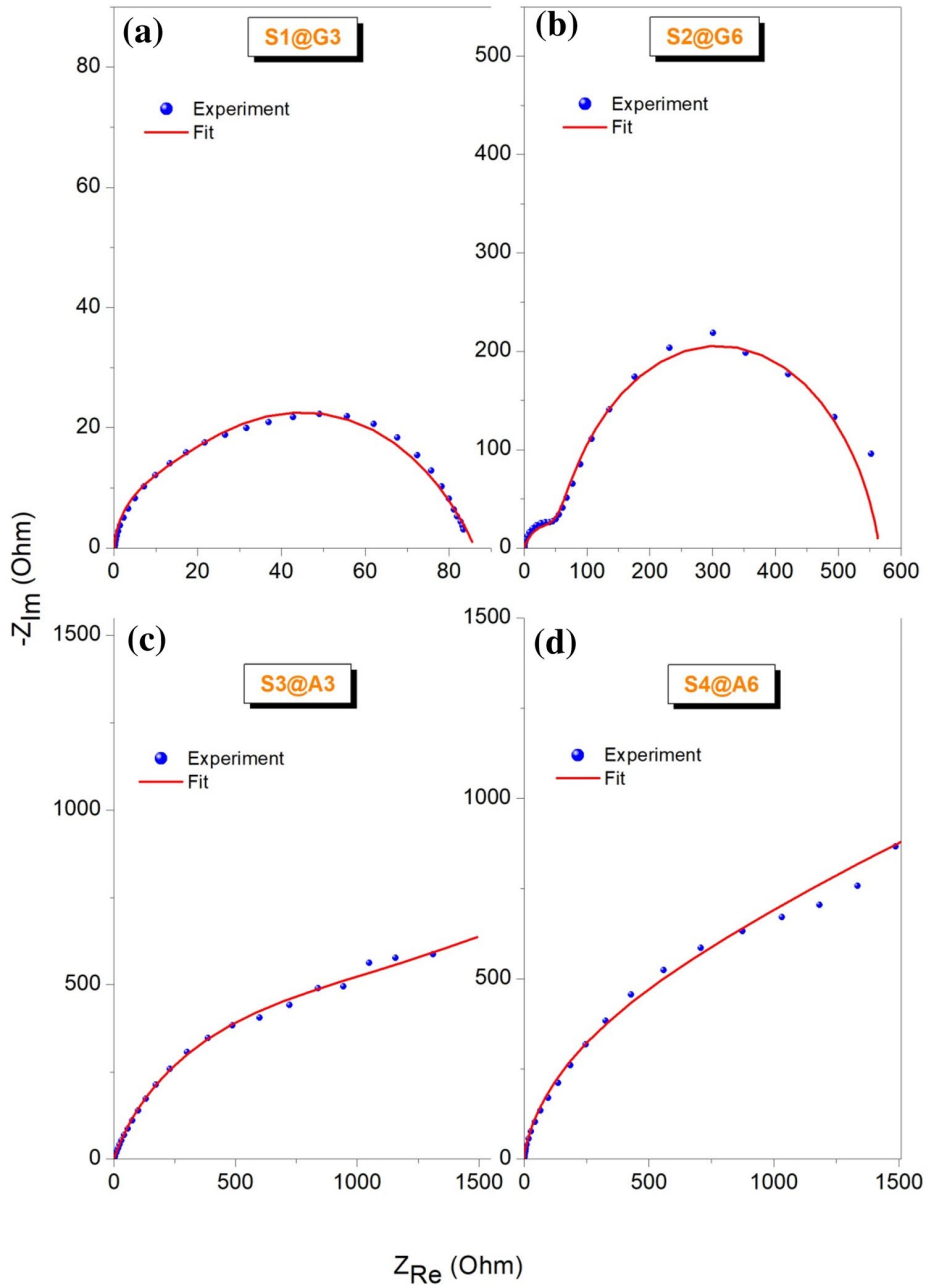
PEIS results of B_4_C materials obtained from the supercapacitor design given in Fig. 2. The red straight lines are the fitted curves obtained from ZFit software. The equivalent circuit and the values of each corresponding circuit elements have been listed in Supp. Material (Fig. S7 and Table S1) which helps fully to deduce the electrode properties.

**Figure 7:**
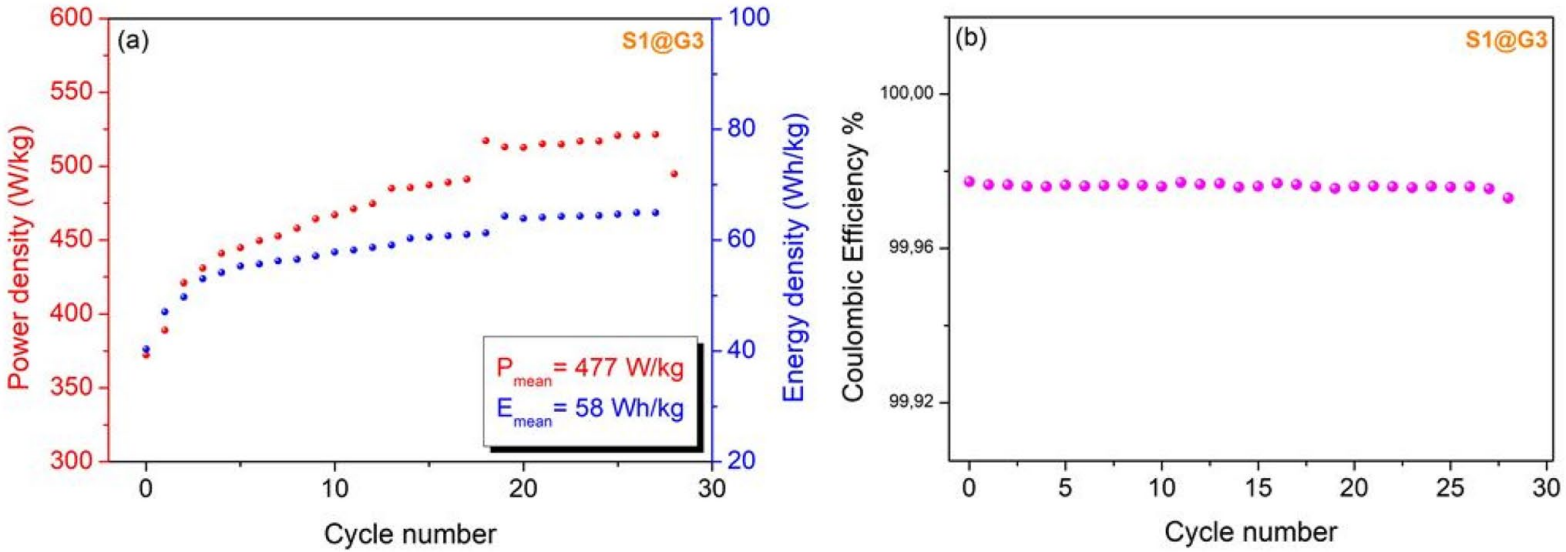
(**a**) Power and energy density, (**b**) Coulombic efficiency for about 30 scans of S1@G3 sample as an electrode. This sample has been synthesized by the graphite and B_2_O_3_ as starting materials and the highest defect concentration obtained from EPR.

Finally, in Figs. 6 and 7 electrochemical performance test of B_4_C materials is presented when they are used as an electrode for the supercapacitor device. The electrochemical performance tests have been applied by following the general guidelines^59,60^ and the equations given in Supplementary Material.

The device design and the components of the supercapacitor can be seen in Fig. 2. According to PEIS results given in Fig. 6a–d the pronounced effect of defect centers can be seen as follows: the equivalent series resistance (ESR) which mostly responsible for the charge transfer is too low compared to the other three designs. Based on the Nyquist plot, the resistance is around 80 Ohm which shows a highly conductive system compared to the ones given in Fig. 6b–d. his supercapacitor also did not reveal any other circuit elements such as Warburg. However, the resistive Warburg element has been obtained for the other three designs indicating at a certain low frequency the mass transport or ion diffusion starts. This also prevents the system to form a complete semi-circle. But in the case of S1@G3 a complete semi-circle has been observed thus the designed supercapacitor has only the ESR and capacitance elements involved in faradaic reactions which gives a typical Randless cell^61^. Whereas, the other three cells have modified Randless cell with Warburg element. Furthermore, this optimized behavior of S1@G3 electrode motivated us to perform GCPL measurements on it and test its specific capacity, energy and power density and, Coloumbic efficiency.

The results show that the B_4_C materials are promising candidates for supercapacitor devices with their high energy density while the major problems of the supercapacitors are their low energy density. In terms of specific capacity, it has been reached around 2000 mAh/g at the first cycles which is a good value for a supercapacitor (refer Fig. S4). The Coulombic efficiency was obtained by the ratio of charging and discharging curves with respect to cycle number and as it is seen in Fig. 7b the efficiency is almost outstanding for the device.

The Raman and EPR results indicate a close relationship between the intrinsic defects, starting materials (synthesis), and electrochemical performance. This has been confirmed by potentiostatic electrical tests.

## Conclusions

In the present study, enhanced characterization techniques were used for identifying the defect centers and their roles in B_4_C material. It has been found that the higher the defect concentration the higher the conductivity. The increase of defect centers is also closely related to starting material but not the B but the C source. Although several peculiarities of B_4_C have been studied so far the defect structures remained somewhat unclear in ceramic society. Moreover, here a supercapacitor device design has been introduced which gives reliable information on the energy storage mechanism of this material system. Performance of supercapacitors composed of various carbonaceous electrodes in terms of their performance parameters such as specific capacitance, energy density and power density values has been tabulated in table given in Supp. Material. The comparison of the reported works and this work reveal that every material has its own characteristic performance depending on numerous factors. Following this, a relatively high energy density (58 Wh/kg) and high specific capacity (2000 mAh/g) and specific capacitance (96.9 F/g at 0.1 A/g) values were obtained meaning that B_4_C is a highly promising material for energy storage purposes. The power density is lower than expected (477 W/kg) but there is plenty of room for this issue to be improved. One of the main strategies might be changing the synthesis route and by this controlling the defect structures and obtaining better performance. Alternatively, doping with metal or rare-earth ion, reducing the crystalline size to nanoscale, or producing B_4_C based composites will be another smart approach to get higher electrochemical performance from B_4_C.

## Supplementary Information

Supplementary Information.
