# Supplementary material for: Defect-induced B4C electrodes for high energy density supercapacitor devices
Source: Sci Rep. 2021 Jun 2;11:11627. doi: 10.1038/s41598-021-90878-0 (PMC8172886; doi:10.1038/s41598-021-90878-0)
Supplement: Supplementary file 1 — Supplementary Information. [file 41598_2021_90878_MOESM1_ESM.pdf]

Supplementary Material

Defect-induced B_4C electrodes for high energy density supercapacitor devices

Özge Balcı[1,2,*], Merve Buldu[3], Ameen Uddin Ammar[3], , Kamil Kiraz[4], Mehmet Somer[1,2,4], Emre Erdem[3,5*]

[1]Koç University Boron and Advanced Materials Application and Research Center, Rumelifeneri Yolu, 34450 Sarıyer, İstanbul, Turkey

[2]Koç University, Department of Chemistry, 34450 Sarıyer, İstanbul, Turkey

[3]Faculty of Engineering and Natural Sciences, Sabanci University, 3495 Tuzla, İstanbul, Turkey

[4]Pavezyum Chemicals Inc., Tuzla, İstanbul, Turkey

[5]Integrated Manufacturing Technologies Research and Application Center & Composite Technologies Center of Excellence, Sabanci University, Teknopark Istanbul, 34906 Pendik, İstanbul, Turkey

*corresponding authors: obalci@ku.edu.tr, emre.erdem@sabanciuniv.edu

Structural Characterization:

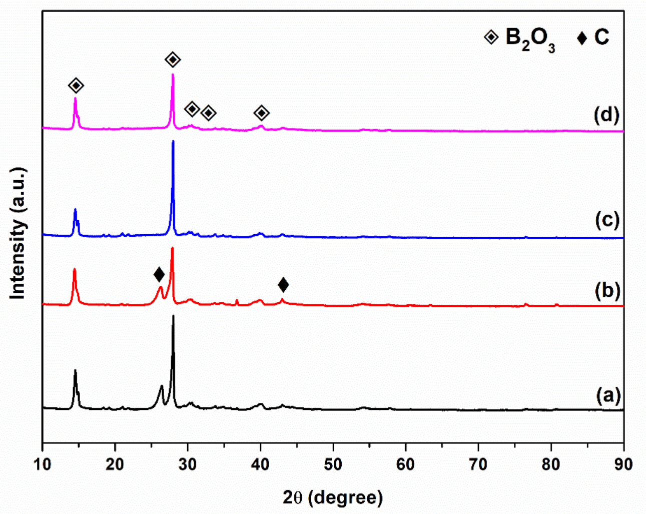

Figure S1: XRD patterns of the mechanically activated powders of anhydrous boron oxide (B_2O_3) and varying carbon (C) sources: (a) graphite, 3 h of milling, (b) graphite, 6 h of milling, (c) active carbon, 3h of milling, and (d) active carbon, 6 h of milling.

Microscopic Characterization:

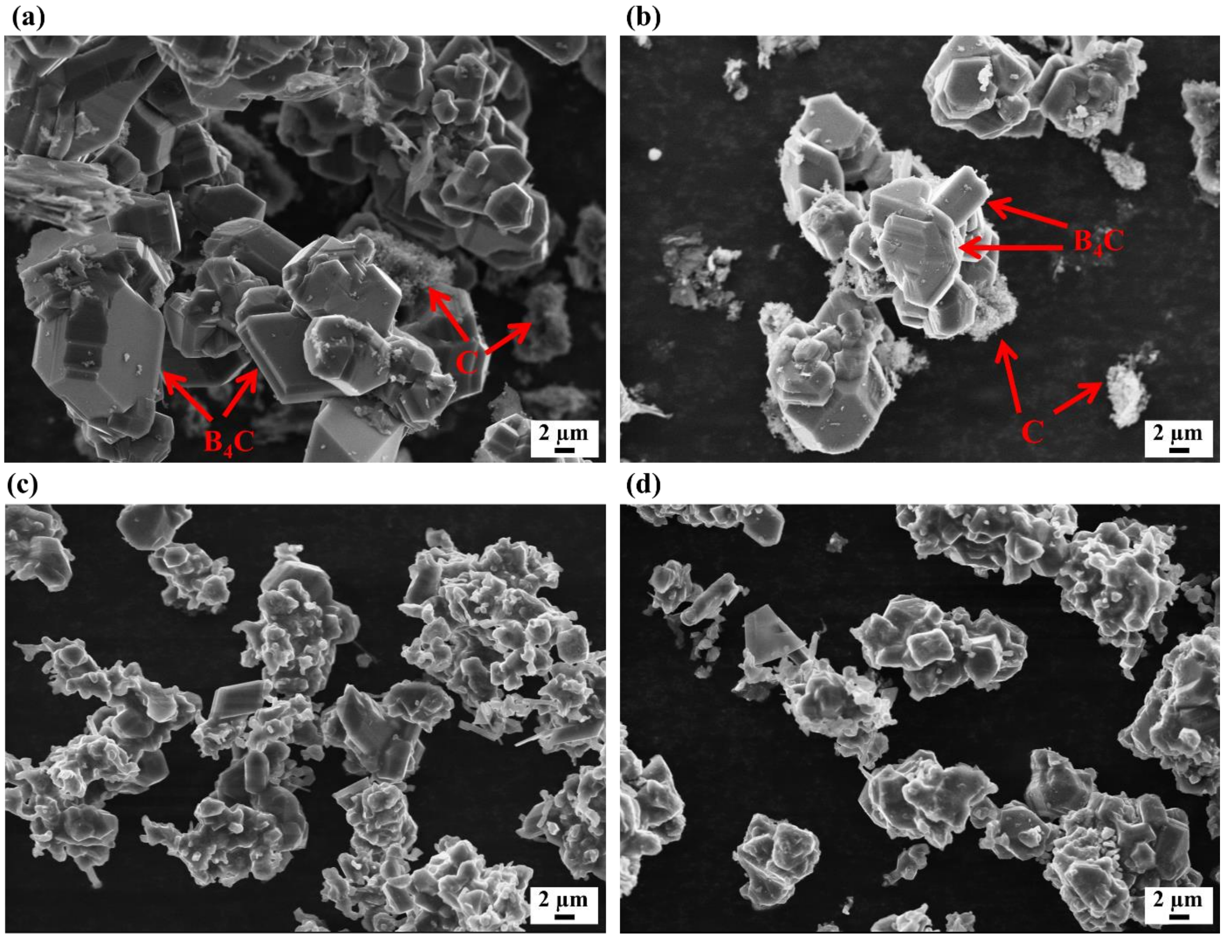

Figure S2: Secondary electron SEM images of the synthesized B_4C powders: (a) S1@G3, (b) S2@G6, (c) S3@A3, and (d) S4@A6.

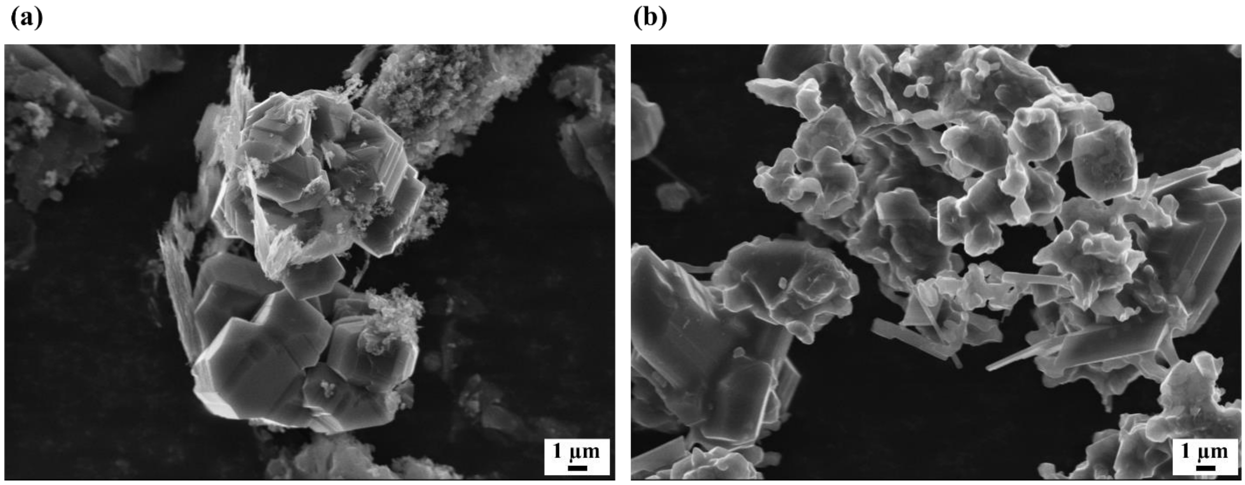

Figure S3: Secondary electron SEM images at high magnifications. B_4C powders synthesized by the graphite (a) S1@G3, and the active carbon (b) S3@A3, as the carbon source.

Thermal Charactrerization:

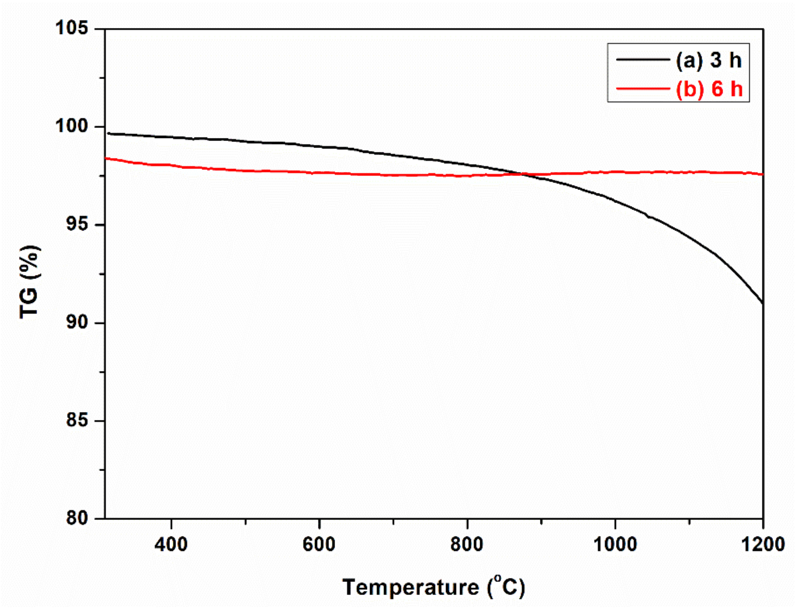

Figure S4: TG analyses of the synthesized B_4C powders: (a) S3@A3, and (b) S4@A6.

Spin counting procedure:

In order to accurately count the number of spins, there are many important issues that should be considered before and after the EPR experiment. Crucial issues, which have to be carefully taken into account, are: (i) Samples should be always weighed before the experiment to avoid complications due to different sample amounts in EPR tubes. If it is not possible to have always the same amount of sample in an EPR tube, then for normalization each spectrum should be multiplied by a filling factor (deduced from the mass of the sample in the EPR tube). (ii) The sample position should be always adjusted to the center of the microwave cavity. (iii) If there is a background EPR signal (e.g., from impurities in the resonator), it has to be subtracted from the EPR signal of the sample. (iv) One should always be careful not to saturate the EPR signal by applying too high microwave power. The microwave phase should be carefully adjusted during the critical coupling (tuning) of

the resonator. (v) One has to check whether there is an offset in the magnetic field and calibrate if necessary. (vi) The *Q* value of the resonator has to be measured and all spectra should be referred to the same *Q* value. The number of defect centers can be quantitatively determined by the aid of EPR spectra independent from the microwave frequency. In order to calculate the defect concentration, one doubly integrates each EPR first-derivative signal. By comparing the integral of the standard sample (here, MnO powder) and the measured sample one obtains the corresponding number of spins, thus the concentration of defect centers. For an exact determination of defect concentration, one has to normalize by taking into account the following expression including experimental parameters of both the reference and the probe under investigation:

$$N_S^* \cdot \frac{RG^*}{RG} \cdot \frac{MF^*}{MF} \cdot \frac{MA^*}{MA} \frac{CT^*}{CT} \cdot \sqrt{\frac{P^*}{P}} \cdot \left(\frac{Scans^*}{Scans}\right)^2 \cdot \left(\frac{SW}{SW^*}\right)^2 \cdot \frac{S^*(S^*+1)}{S(S+1)} = corrected\ value \qquad (1)$$

where N_S^*, *RG, MF, MA, CT, P, Scans, SW, and S* stand for the number of spins in reference sample, receiver-gain, modulation frequency (in kHz), modulation amplitude (in G), conversion time (in ms), microwave-power (in mW), field-sweep (in G), number of scans, and spin quantum number, respectively. Note that, (*) indicates the measurement parameters for the reference sample. Once the normalised-corrected value of N_S^* is obtained, via simple cross multiplication of the N_S^* and the area under the EPR signals reveal the defect concentration, N_D, of the sample under investigation:

$$N_D = \frac{(Area)_D \cdot N_S^*}{(Area)^*} \qquad (2)$$

where $(Area)_D$ and N_D are the area of the EPR signal of the related defect centre and the number of spins of the sample, respectively. In this work we used MnO powder as standard sample, which has 1.75×10^{15} spins/g.

Equations for the Electrochemical Performance Parameters:

The performance properties of the supercapacitors such as specific capacitance, energy density, power density are calculated from the discharge part of the GCPL

curves. The capacitance, C (F) can be obtained by the well-known Equation (3) when calculated from GCPL:

$$C = \frac{I\Delta t}{\Delta V} \qquad (3)$$

where I (A) is the applied current, ΔV (V) is the discharge voltage window excluding the IR drop, Δt (s) is the corresponding discharge time. Specific capacitance, C_m (F·g^{-1} for gravimetric specific capacitance) for the supercapacitor cell is calculated by Eq. (4)

$$C_m = \frac{C}{S} \qquad (4)$$

where S is the mass (g) of the active materials in supercapacitor. The energy density, power density and power density are calculated from GCPL results. Gravimetric energy density, E (W·h·kg^{-1}), is calculated using Eq. (5)

$$E = \frac{C\Delta V_{max}^2}{2m} \cdot \frac{1000}{3600} \qquad (5)$$

where ΔV_{max} (V) is the maximum voltage region applied and m (kg) is the total mass of the active material in both electrodes. The power density, P (W·kg^{-1}) is calculated according to Eq (6):

$$P = \frac{E3600}{\Delta t} \qquad (6).$$

And finally the the coulombic efficiency (%) is calculated by Eq. (7) as follows:

$$Coulombic\ Efficiency\ (\%) = \frac{t_d}{t_c} \cdot 100\% \qquad (7)$$

where t_d and t_c are the discharging and charging times, respectively.

Additional Electrochemical experiments:

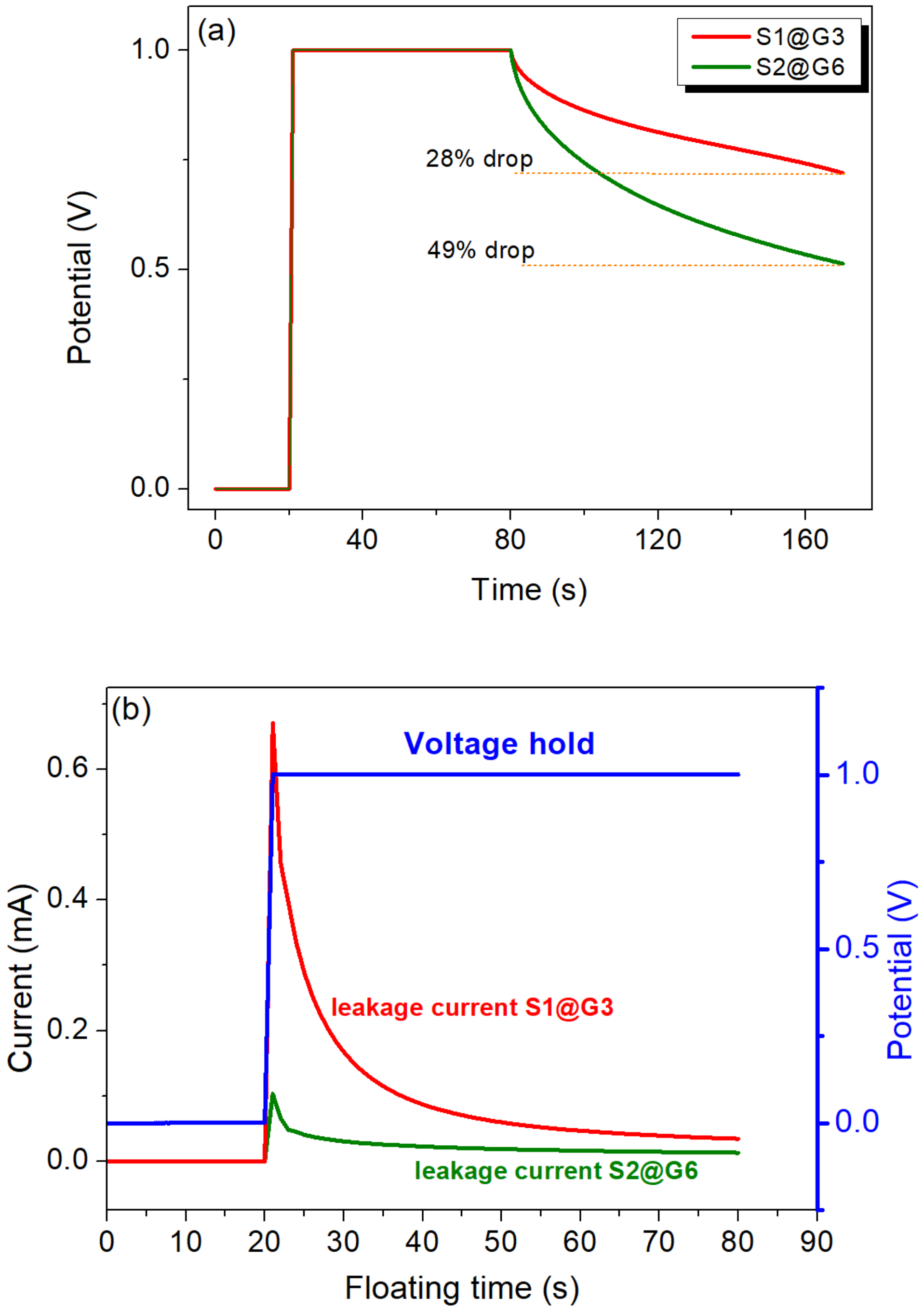

Figure S5: (a) Voltage holding test for two kinds of supercapacitors, namely for S1@G3 and S1@G6. (b) Leakage current profile of S1@G3 and S1@G6 supercapacitor device at 1 V volt holding voltage obtained from the floating test.

From the voltage holding and floating experiments it is obviously clear that the more defective sample (S1@G3) *-according to EPR-* has the less impact on the capacitance than the S1@G6. Since B_4C is highly new type of material for supercapacitor designs as electrode, the voltage hold and leakage performance is highly promising. Of course, we are aware of the results are not as impressive as graphene or metal oxides electrodes, however in next studies, we believe the results will be improved substantially by further modification of materials such as synthesizing nanosized B_4C or producing composite of B_4C with other kinds of energy materials as well as metal ion doping.

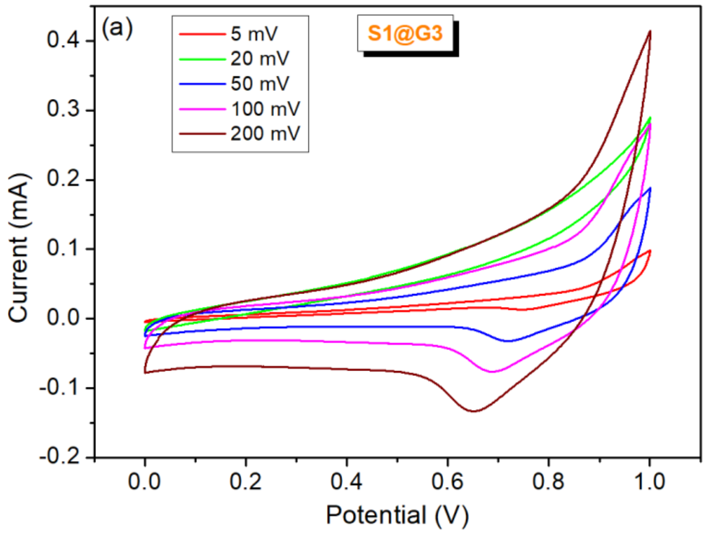

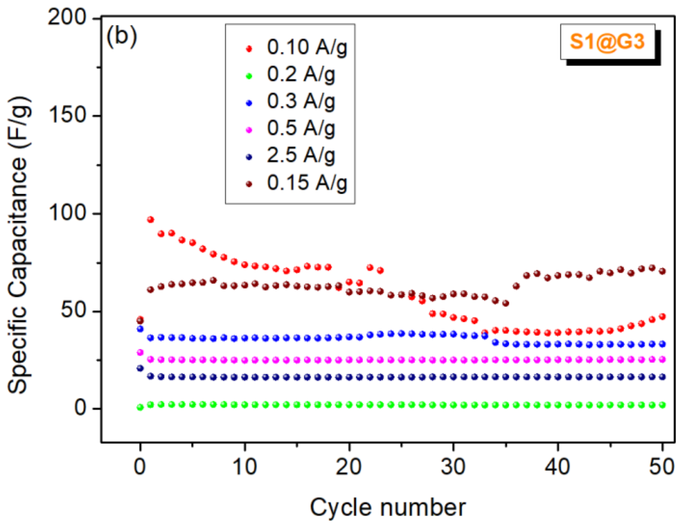

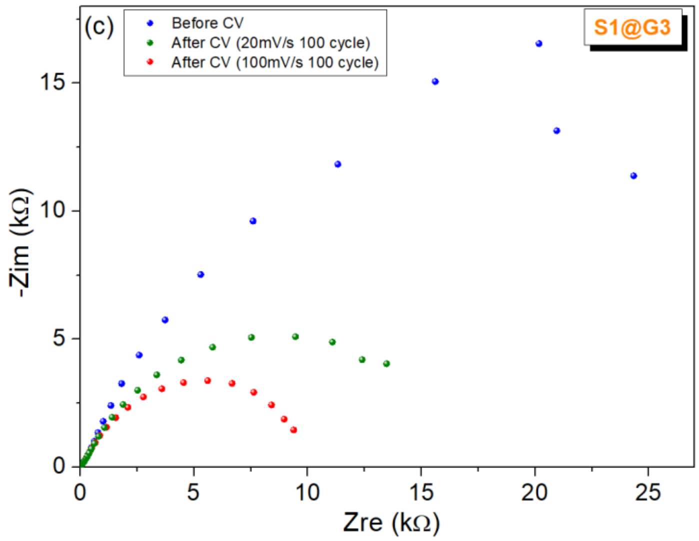

Figure S6: (a) CV measurements at various scan rates, (b) rate capability of S1@G3 obtained from GCPL measurements (c) Nyquist plots obtained from PEIS measurements before and after 100 CV cycles with different scan rates at 20 mV/s and 100 mV/s.

Supercapacitor from the electrode of S1@G3 has been thoroughly tested by CV, GCPL and PEIS as esteemed Reviewer suggested and the results are presented in Fig. S6 (a-b-c). From such results basically the supercapacitor performance is quite promising but not outstanding. CV curves measured at 5 mV/s up to 200 mV/s in Fig. S6(a) is almost identical even at higher scan rates confirming the good electrochemical stability of the electrodes. The shape with presence of redox humps in the CV curves confirms the battery-like pseudocapacitive mechanism. On the other hand, the specific capacitance at the highest specific current rate was obtained as 96.9 F/g at 0.1 A/g, respectively which was obtained by considering the Eq. 4. Also Nyquist plot were plotted both before and after 100 CV scans and the differences are not much while anyway the impedance values are in the range of kOhm. Nevertheless, after 100 cycle the impedance become much smaller which shows the more the cycle time the more the electrons will transfer and find easier path for conduction. Similar results have been reported for supercapacitors composed of NiO nanofiber electrodes and the effect has been explained by the possible loss of adhesion of some active materials with the current collector during the cycling[1].

In order to fully understand the properties of the electrodes in designed supercapacitors all Nyquist plots obtained from PEIS were fitted to suitable equivalent circuits by the aid of ZFit.

Supp. Table 1: Equivalent circuit elements and their numerical values obtained from fitting of the Nyquist plots given in Fig. 6 in the main text.

	R1 (Ohm)	R2 (Ohm)	R3 (Ohm)	C2 (μF)	Q1 (μFa$_1^{-1}$)	Q2 (μFa$_1^{-1}$)	Q3 (μFa$_1^{-1}$)	a1	a2	s2 (Ohm·s$^{1/2}$)
**S1@G3**	1000	100	10	0.1	-	-	5	-	-	1
**S2@G6**	510	55	-	-	8	5	-	0.86	0.7	-
**S3@A3**	27288	600	-	-	128	800	-	0.72	0.27	-
**S4@A6**	10603	0.4	-	-	303	11	-	0.35	0.97	-

The main circuit elements are designated as follows:

R: Resistor, the simplest element. It only has the real values and independent of the frequency. In case of Randless circuit R1 stands for the series resistance of the electrolyte, C is and R2 is the charge-transfer resistance.

C: Capacitor element mostly for double layer.

Q: Constant phase element (CPE). This element is frequency dependent part of the capacitance.

a and *s* are the crucial internal parameters/units to obtain good fit.

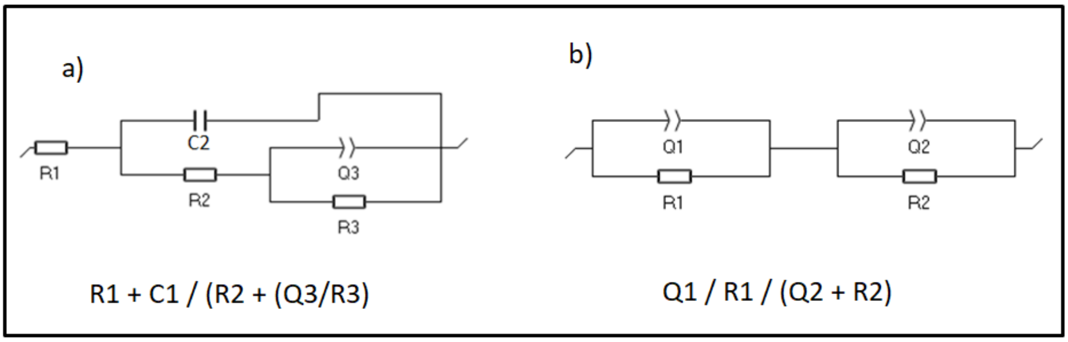

Figure S7: The equivalent circuits that are obtained by the aid of Zfit software. a) Equivalent circuit for S1@G3 and, b) equivalent circuit for S2@G6, S1@A3 and, S1@A6. Refer Fig. 6 in main text for the fitted curves.

Specific Capacity vs potential results obtained from GCPL test

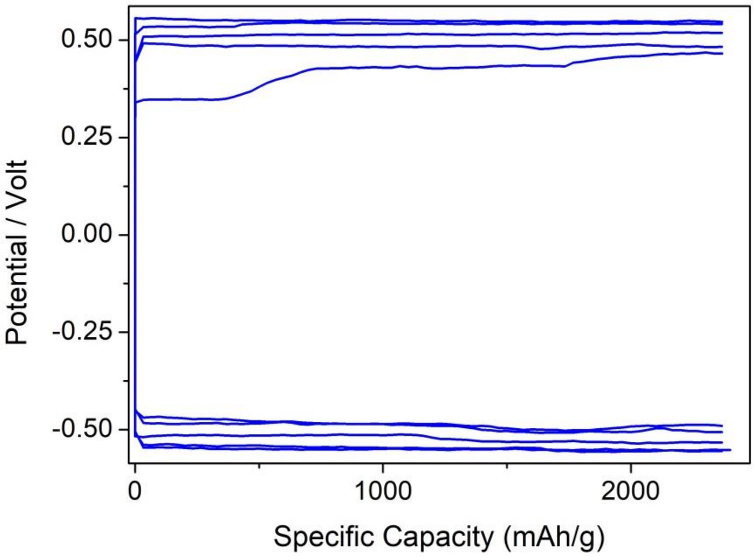

Figure S8: Specific capacity revealing more than 2000 mAh/g at first cycles.

Performance comparison with other carbonaceous materials

A compact table listing the specific capacitance, power and energy density values of other carbon-based materials compared to this work.

Supp. Table 2: Electrochemical performance of this work and various carbon-based electrodes used in supercapacitor devices.

Electrodes	Specific capacitance (F/g)	Power Density (W/kg)	Energy Density (Wh/kg)	Reference
B_4C / Active C	96.9	477	58	This work
Graphene Oxide / Hydrogen Annealed Graphene	306	148	41	[2]
Carbonized soybean residue activated carbon (symmetric)	489	1000	69	[3]
3D carbon frameworks (symmetric)	174	150000	34	[4]
Hexagonal boron nitride nanosheet / carbon nanocomposite	250	245	17	[5]

References:

1. M. Zhang, Q. Li, D. Fang, I. A. Ayhan, Y. Zhou, L. Dong, C. Xiong and Q. Wang, *RSC Advances*, 2015, **5**, 96205-96212.
2. H. Yang, S. Kannappan, A. S. Pandian, J. Jang, Y. S. Lee and W. Lu, *J. Power Sources*, 2015, **284**, 146-153.
3. H.-Y. Chung, G.-T. Pan, Z.-Y. Hong, C.-T. Hsu, S. Chong, T. C. Yang and C.-M. Huang, *Molecules*, 2020, **25**.
4. C. Leng, Z. Zhao, Y. Song, L. Sun, Z. Fan, Y. Yang, X. Liu, X. Wang and J. Qiu, *Nano-Micro Letters*, 2020, **13**, 8.
5. T. Li, X. Jiao, T. You, F. Dai, P. Zhang, F. Yu, L. Hu, L. Ding, L. Zhang, Z. Wen and Y. Wu, *Ceramics International*, 2019, **45**, 4283-4289.
